# Sleep disturbances in adults with chronic kidney disease: an umbrella review

**DOI:** 10.1007/s40620-025-02214-8

**Published:** 2025-02-08

**Authors:** Ginger Chu, Lisa Matricciani, Sarah Russo, Andrea K. Viecelli, Shilpanjali Jesudason, Paul Bennett, Ritin Fernandez

**Affiliations:** 1 School of Nursing and Midwifery, University of Newcastle, University Drive, Callaghan, NSW 2305 Australia; 2 Clinical and Health Sciences, University of South Australia, Adelaide, Australia; 3 Alliance for Research in Exercise, Nutrition and Activity (ARENA), University of South Australia, Adelaide, Australia; 4 Nephrology Department, John Hunter Hospital, New Lambton Heights, NSW Australia; 5 Australasian Kidney Trials Network, University of Queensland, Brisbane, Australia; 6 Faculty of Medicine, University of Queensland, Brisbane, Australia; 7 Department of Kidney and Transplant Services, Princess Alexandra Hospital, Brisbane, Australia; 8 Central Northern Adelaide Renal and Transplantation Service, Royal Adelaide Hospital, Adelaide, Australia; 9 School of Nursing and Midwifery, Griffith University, Nathan, QLD Australia

**Keywords:** Chronic kidney disease, Sleep, Sleep disturbances, Reviews

## Abstract

**Background:**

This umbrella review aimed to synthesise the existing evidence on sleep disturbances and sleep disorders in the adult chronic kidney disease (CKD) population.

**Methods:**

A systematic search across five electronic databases. Reviews were grouped according to aspects of sleep and the focus of the review. The JBI critical appraisal checklist was used for quality assessment, and Preferred Reporting Items for Overviews of Reviews (PRIOR) guideline was used for reporting. The protocol was registered in the international registry PROSPERO (CRD42024527039).

**Results:**

We identified 50 reviews covering three main aspects of sleep (sleep apnoea, restless legs syndrome and other sleep disturbances) across five focus areas (prevalence, interventions, health outcomes, determinants of sleep and patient experience). Most reviews reported on sleep disturbances (72%, 36 reviews) and focused on interventions (58%, 29 reviews). In contrast, evidence on sleep determinants and patient experience was limited. A high prevalence of sleep apnoea (49%), restless legs syndrome (27.2%) and other sleep disturbances (55%) was reported. Non-pharmacological interventions, including aromatherapy, dialysis, muscle relaxation, yoga, music, and nurse-led management, were found to improve sleep. However, this evidence was based on a single meta-analysis with few primary studies.

**Conclusions:**

Despite the growing number of reviews on interventions to improve sleep, the evidence for their effectiveness is limited by the small number of primary studies and the high degree of overlap between reviews. Further research is needed to identify effective interventions. Additionally, qualitative studies exploring patients’ perspectives on sleep are essential, as evidence in this area remains scarce.

**Graphical abstract:**

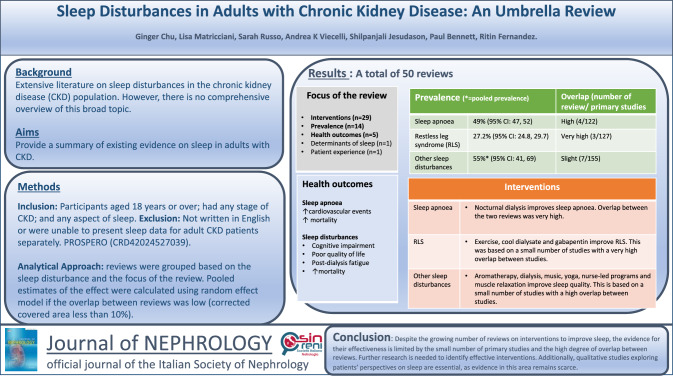

## Introduction

Chronic kidney disease (CKD) is a significant health issue, affecting more than 10% of the global population, accounting for approximately 800 million individuals worldwide [2]. Individuals with CKD experience a high symptom burden, poor quality of life, and increased morbidity and mortality [3]. One significant risk factor associated with mortality and impaired quality of life of people with CKD is poor sleep [4].

Sleep is important in maintaining healthy kidney function and the overall well-being of individuals [5]. An 11-year longitudinal study of 4238 individuals found that short sleep duration is linked to a rapid decline in kidney function [6]. Furthermore, individuals with CKD who experienced reduced sleep and increased arousal per hour of sleep face a 50% higher risk of mortality [7] and report a poorer quality of life [8], compared with those with less sleep disturbances.

Disturbed sleep may present as a single or overlapping sleep disorder with different pathophysiological mechanisms and implications for outcomes (e.g., fatigue or mortality). The causes of poor sleep among people with CKD are often multifactorial, involving complex intrinsic factors such as uraemia and extrinsic factors such as dialysis treatment [9]. Many hypotheses have been proposed, for example, uraemic encephalopathy [10] may cause excessive daytime sleepiness and change day/night sleep patterns. Additionally, the abnormal production and regulation of melatonin due to reduced kidney function can impact circadian rhythms and lead to sleep disturbances [11]. Extrinsic factors, such as dialysis-related treatment, including dialysis noise [12] and the effect of dialysate temperature [13] have all been associated with sleep disturbances.

The significance of sleep disturbances has led to the publication of many systematic reviews and meta-analyses over the years, aiming to understand the prevalence [14–17], treatment options [18–21], and implications for outcomes [22–24] in the CKD population. An overview of the reviews is needed to provide a comprehensive landscape of this broad topic, compare the findings, and analyse the knowledge gap. The aim of this umbrella review was to provide a summary of existing evidence on sleep in adults with CKD from systematic reviews. This review will offer a bird's-eye view of this broad topic and present and provide a comprehensive picture of the quality of evidence. This information can support the development of guidelines for practice and priority areas for future research.

## Methods

### Study design

An umbrella review of systematic reviews was conducted in accordance with the Preferred Reporting Items for Overviews of Reviews (PRIOR) guideline [25]. The protocol was registered in the international registry PROSPERO (CRD42024527039).

### Search strategies and definitions

Five electronic databases, MEDLINE, EMBASE, Cumulative Index to Nursing and Allied Health Literature (CINAHL), Epistemonikos, and Cochrane Library, were searched in February 2023 and updated again in January 2024. The search strategy combined subject headings (MeSH) through Boolean operators “AND” and /or “OR”. The following headings were applied: “kidney disease”, “renal dialysis”, “haemodialysis”, “sleep”, “insomnia”, “sleep disorders”, and “sleep disturbances”. A complete list of search terms is included in Supplementary Table 1.

### Inclusion and exclusion criteria

Studies were included if they were a systematic review that examined (1) participants aged 18 years or over, (2) participants who had any stage of CKD, and (3) any aspect of sleep. Excluded were systematic reviews that were not written in English or were unable to present sleep data for adult CKD patients separately.

### Review selection

Two reviewers (GC & SR) independently screened the titles and abstracts to determine the eligibility of the study. The same reviewers assessed the full text of selected reviews independently in detail against the inclusion criteria. Any disagreements between the reviewers at each stage of the selection process were resolved through discussion or with a third reviewer (LM). Excluded reviews and the reason for exclusion are listed in Supplementary Table 2.

### Data extraction

The lead author (GC) extracted data. To ensure accuracy and consistency, another reviewer (RF) extracted data from 10% of the included reviews (*n* = 5). A spreadsheet was created to chart the following information that contributed to addressing the research aims: author and publication year, aspects of sleep, review focus (e.g., prevalence or intervention), review population characteristics, outcome measures, and the author’s conclusion.

### Methodological quality assessment

The methodological quality of systematic reviews was independently evaluated by three reviewers (GC, LM and RF) using the JBI critical appraisal checklist for systematic reviews [26]. This tool includes 11 questions; each question is scored as 1 (the criteria being met), 0 (criteria not met), 0.5 (criteria is unclear), or “not applicable.” Any disagreement between the reviewers was resolved through discussion. The total score ranged from 0 to 11, with higher scores indicating a better quality of evidence.

### Overlapping analysis

The overlap of primary studies in the included systematic reviews was assessed by the GROOVE (Graphical Representation of Overlap for OVErviews) [27] methodological approach. This tool provides a matrix that calculates the degree of overlap, presented as a corrected covered area (CCA) and categorised as slight overlap (CCA < 5%), moderate overlap (CCA 5–10%), high overlap (CCA > 10–15%) and very high overlap (CCA > 15%). The overlap analysis was performed for each aspect of sleep based on the review focus. The integration of CCA in the result interpretation was followed by the guidance for using CCA in meta-reviews [28].

### Data synthesis

Inter-rater reliability (IRR) for title/abstract screening and full-text screening was measured using Cohen’s kappa coefficient (k) generated from Covidence [29]. Reviews were grouped according to aspects of sleep and the review focus. Due to the complex sleep parameters, for this review, aspects of sleep were categorised into sleep disturbance (defined in terms of reduced sleep quantity or quality [30, 31]) and other medical diagnoses of sleep disorders. Sleep disorders, such as restless legs syndrome (RLS) or sleep apnoea, were reported as defined in the included systematic reviews. Where there was more than one focus (for example, a review that reported both prevalence and intervention), the primary focus was prioritised based on the aim of the review. A statistical meta-analysis was performed using STATA (version 18) [32]. A random effects model employing the Freeman- Tukey-Transformed proportion was used to measure the effect size. Pooled effect sizes were expressed as proportions with 95% confidence intervals (CI) calculated using restricted maximum likelihood approach. To avoid overestimating the effect size, if the CCA was more than 10% (high overlap), the pooled effect was calculated by including non-overlapped meta-analyses and one of the overlapped meta-analysis in the following order: Cochrane review, the most recent or highest quality meta-analysis [33]. If all meta-analyses were overlapped, the effect size was reported from a meta-analysis in the same order as above.

## Results

**Fig. 1:**
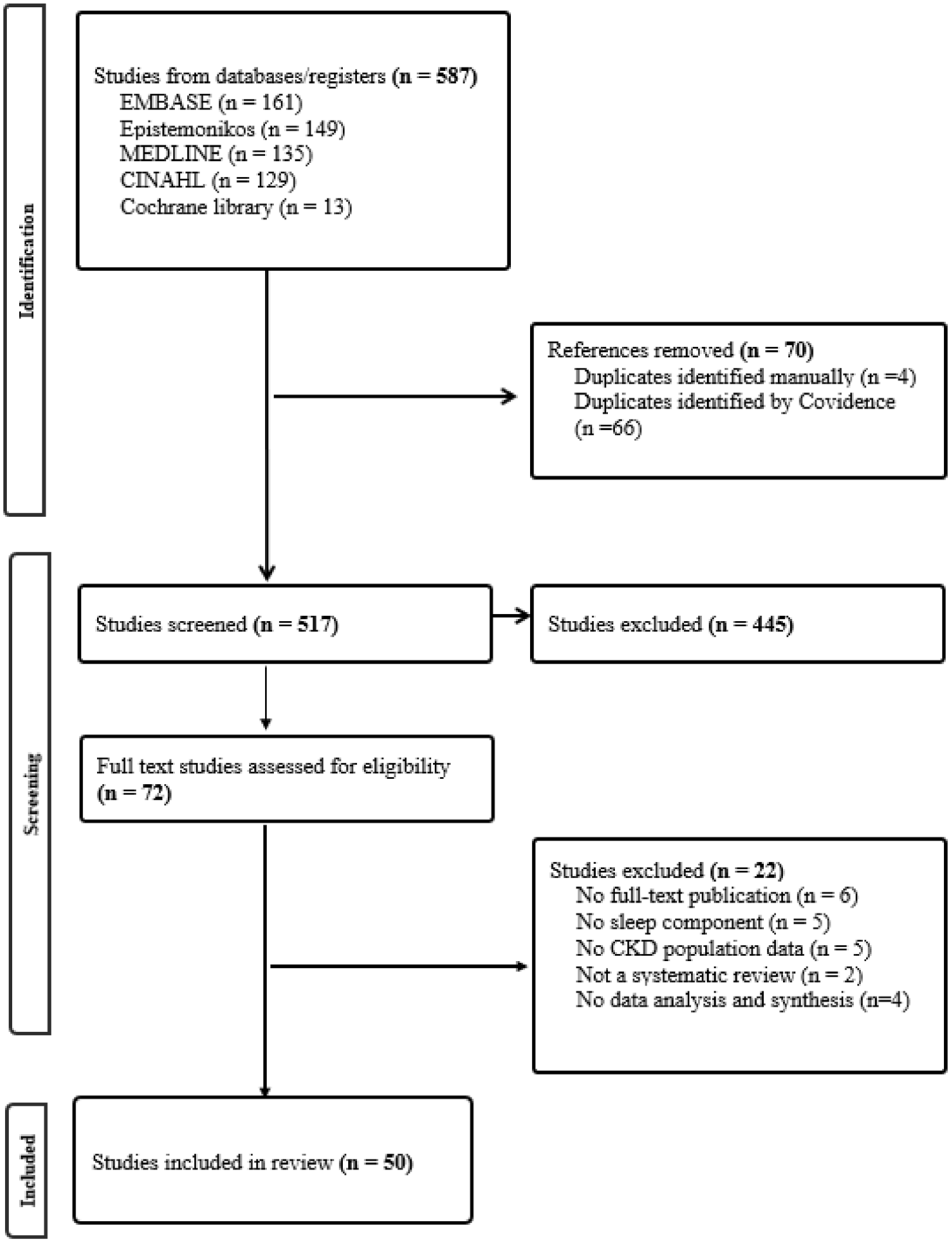
Flow diagram indicating selection of articles

After removing duplicates from 587 reviews, 517 remained for title and abstract assessment, and 72 were retrieved for full-text review. Finally, 50 systematic reviews met the eligibility criteria and were included in the quality assessment. The IRR for title/abstract screening and full-text screening was* k* = 0.58001 and * k*  = 0.75294, respectively. Figure 1 outlines the flow of searches through the inclusion process.

### Study quality

The critical appraisal scores of the 50 reviews ranged from 3 to 11; the median (interquartile) score was 11 (9.5, 11). Thirteen reviews (26%) did not assess the potential presence of publication bias and its impact on the results. Ten reviews (20%) either did not have evidence or were unclear regarding independent appraisal. Seven reviews (14%) either did not have evidence or were unclear regarding the study combination. A summary of the methodological quality of each criterion from the included reviews is presented in Table 1.

**Table 1:**
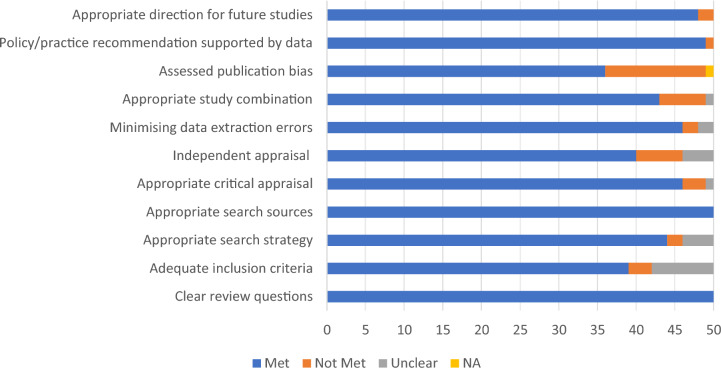
Summary of the methodological quality of each criterion from the included reviews

### Characteristics of included reviews

Table 2 reports the characteristics of the reviews included in this umbrella analysis. The 50 included reviews were published between 2007 and 2024, and 33 included a meta-analysis.

**Table 2:** Summary of included reviews

Study	No. primary studies* (*n*)	Stage of CKD	Instruments	Outcomes	
**PREVALENCE**					
***Other Sleep Disturbances***					
Rehman, 2019	28* (54,868)	HD	NR	Sleep disturbances in HD: 40% (95% CI 30, 49, *I*^2^: 99.8%)	
Fletcher, 2022	48* (11,012)	Mixed	AIS, CKD-SBI, CKD-SI, CSE, DSI, LUSS, MSAS-SF, POS-Renal, PSQI	Poor sleep symptoms in CKD without KRT (49%, 95% CI 41, 58); Dialysis (57%, 95% CI 52, 62); Transplantation (31%, 95% CI 14, 48)	
Mirghaed, 2019	21* (9,432)	HD	NR	Poor sleep quality in HD: 75.30% (95% CI 70.08, 82.50, *I*^2^: 50.3%)	
Tan, 2022	93* (45,796)	Mixed	PSQI, ICSD	Poor sleep quality^a^: overall CKD: 64% (95% CI 59, 68, *I*^2^:97%); CKD without KRT: 59% (95% CI, 44, 73, *I*^2^: 98%), HD: 68% (95% CI, 64,73, *I*^2^*:* 95%), PD: 67% (95% CI, 44, 86, *I*^2^: 97%); transplantation 46% (95% CI, 34, 59, *I*^2^: 96%). Insomnia^b^: overall CKD: 45% (95% CI 38, 51,* I*^2^:99%); CKD without KRT: 48% (95% CI, 30, 67, *I*^2^: 94%), HD: 46% (95% CI, 39, 54, *I*^2^*:* 98%), PD: 61% (95% CI, 41,79, *I*^2^: 91%), and Transplantation: 26% (95% CI, 9, 49, *I*^2^: 97%)	
Ren, 2019	4	CKD > 60	SF-36, DSI, Euroqol, EQ-5Q-5L, MSAS-SF	Poor sleep quality: > 40%	
Clark-Cutaia, 2022	10	HD	POS-Renal, POS-Renal (Spanish) Brief COPE, DSI, DASS-21, ESAS, KDQoL-36, PSQI, SF-36, WHO-QOL-BREF	Sleep disturbance was one of the most prevalent symptoms in the HD population (summary of 10 studies)	
Murtagh 2007	17	KRT	PSQI, self-reported	Sleep disturbances: 44% (range: 20, 83)	
***Sleep Apnoea***					
Huang 2019	26* (90,058)	CKD not on dialysis	ESS, PSQI, ICD-9, PSG, type 3 monitor, SDQ	Sleep apnoea: 38% (95% CI 21–70, *I*^2^: 100%)	
Pisano 2024	107* (140,279)	Mixed	Sleep questionnaires, PSG, type 3 monitor	Sleep apnoea: CKD not on dialysis (57%, 95% CI 42–71,* I*^2^: 99.4%); ESKD 49% (95% CI 47–52,* I*^2^: 99.9%)	
Hansrivijit 2021	16* (340,587)	Mixed	PSG, ICD-9	Sleep apnoea in CKD: 47.5% (95% CI 28.8–66.9, *I*^2^*:* 98.9%)	
Nigam 2016	8	Mixed	PSG	CSA in CKD is 9.6%	
***RLS***					
Zhou 2023	97* (23,248)	HD	IRLSSG	RLS: 27.2% (95% CI 24.8, 29.7,* I*^2^: 95.6%) RLS: Overall CKD: 24.2% (95% CI, 20.1, 28.7,* I*^2^: 96.1%). Dialysis (28.4%, 95% CI, 24.6–32.6), Early stages CKD: 9.9% (95%CI, 5.4, 17.5), Transplantation: 6.7% (95% CI, 5.6, 7.8)	
Lin 2016	51* (12,932)	Mixed	IRLSSG, interviews		
Ghanei Gheshlagh 2017	26* (6,188)	HD	NR	RLS: 50% (95% CI 38–61) in Iranians and 30% (95% CI 23–37) in international databases	
**INTERVENTIONS**					
***Aromatherapy***					
Zhang, 2023	6* (400)	HD	PSQI, VAS	Sleep quality improved by aromatherapy (SMD: – 1.52, 95% CI – 2.38, – 0.67,* I*^2^: 93.2%)	
Bouya, 2018	3	HD	PSQI	Sleep quality improved by aromatherapy	
Yang 2020	2	HD	PSQI, VAS	Sleep quality improved by inhaled aromatherapy	
***Acupressure***					
Pei, 2021	8* (618)	HD	PSQI	Auricular acupressure may be an alternative treatment for insomnia in patients with HD (acupressure vs estazolam: MD: – 0.64, 95% CI – 3.86, 2.57,* I*^2^: 92%)	
Wang, 2020	9* (626)	HD	PSQI	Acupressure improves sleep quality (SMD:—0.81, 95% CI —1.26,—0.36,* I*^2^: 78.6%)	
Yang, 2022	6* (399)	HD	PSQI	Acupressure improves sleep quality (MD = -1.97, 95% CI – 2.62, – 1.32, *I*^2^: 43%)	
Yang, 2015^+^	3* (211)	KRT	PSQI, PSG, SF-36, VAS	Sleep quality improved by acupressure (SMD: 1.77, 95% CI 0.80, 2.73)	
Natale, 2019^+^	6* (367)	Mixed	PSQI	It is very uncertain whether acupressure makes any difference to sleep quality (MD-1.27, 95% CI – 2.13, – 0.40)	
Kim, 2010	3	Mixed	PSQI, sleep log, SF-36, VAS, Self-rating scale of sleep, Rate of sleep-disturbance-rated complaints	No definitive conclusion on the effect of acupressure on sleep quality due to the heterogeneity of studies	
Chu, 2022^+^	6	Mixed	Sleep quality: PSQI, VAS	Sleep disturbances are alleviated (reduced by 6.2–50%) by acupressure	
***Acupuncture***					
Kim, 2016	4* (180)	Mixed	PSQI	No definitive conclusion impact of acupuncture on sleep quality due to low-quality evidence	
Melo 2020	4	Mixed	PSQI	Sleep quality improved by acupuncture	
***Dialysis***					
Budhram, 2020	4	KRT	KDQoL, Choice Health Equality Questionnaire, Sleep Problem Index	Compared with PD, In-centre HD was associated with better sleep quality (no effect size reported)	
Kennedy 2018	16* (837)	KRT	PSG, Actigraphy, MSLT, MOS-SPI II, ESS, IRLS, SF-36, Interviews	Sleep quality improved by intensity KRT (either intensive HD, CCPD or transplant) (RR: 0.53; 95% CI 0.44, 0.64, *I*^2^: 57%)	
Lavoie, 2019	4* (91)	HD	PSG (AHI, mean SpO_2_)	Sleep apnoea improved by NHD (MD: – 11.9, 95% CI – 13.47, – 10.37,* I*^2^: 0%)	
Li, 2018	5* (62)	KRT	PSG	Sleep apnoea improved by NHD (MD: – 14.90, 95% CI – 20.12, – 9.68, *I*^2^: 60.4%)	
***Exercise***					
Zhang 2022	19* (989)	Mixed	ESS, KDQoL-SF, PSQI, Accelerometer sleep recorder	Sleep quality may be improved by exercise (SMD: − 0.16, 95% CI − 0.62, 0.31, *I*^2^: 87%)	
Valera, 2024	11* (130)	Mixed	PSQI, Leicester uraemic symptom scale, sleep diary, ESS, Massachusetts sleep diary, accelerometry	Sleep quality improved by exercise (MD: – 5.72, 95% CI – 7.76, – 2.77, *I*^2^: 93%)	
Yang, 2015^+^	1* (28)	KRT	PSQI, PSG, SF-36, VAS	Sleep quality improved by exercise (SMD: 3.36, 95% CI 2.16, 4.57)	
Natale, 2019^+^	4* (138)	Mixed	PSQI	It is very uncertain whether exercise makes any difference to sleep quality (MD: – 1.10, 95% CI – 2.26, 0.05)	
Song, 2018	4* (141)	HD	IRLSSG	RLS improved by exercise (SMD: – 1.79, 95% CI – 2.21, – 1.37, *I*^2^: 0%)	
Gopaluni, 2016	2* (48)	Mixed	IRLSSG	RLS improved by exercise (MD: – 7.56, 95% CI – 14.20, – 0.93, I^2^: 65%)	
***Music***					
Yangoz, 2022	3* (494)	HD	PSQI	Sleep quality improved by music (Hedge's g: 1.95, 95% CI 0.92, 2.97)	
***Muscle relaxation***					
Yang, 2021	6* (494)	HD	PSQI	Sleep quality improved by progressive muscle relaxation therapy (MD: – 1.69, 95% CI – 1.95, – 1.42)	
Natale, 2019^+^	4* (291)	Mixed	PSQI	It is very uncertain whether relaxation makes any difference to sleep quality (MD: – 1.62, 95% CI – 5.03, 1.79)	
***Mindfulness-based interventions***					
Yang, 2015^+^	3* (184)	KRT	PSQI, PSG, SF-36, VAS	Sleep quality is no different in CBT group (SMD: 0.44, 95% CI —0.28, 1.17)	
Razzera 2021	2	HD	PSQI, GHQ-28	No definitive conclusion on impact of mindfulness-based interventions on sleep quality (1 primary study showed a significant improvement, and another did not show significant improvement)	
Nopsopon, 2021	1	PD	PSQI	Sleep quality is no different on CBT + sleep hygiene vs sleep hygiene	
***Yoga***					
Bayülgen, 2022	1	HD	PSQI, VAS	Yoga was reported to be effective on sleep disorders	
Chu, 2022^+^	3	Mixed	Sleep quality: PSQI, VAS	Sleep disturbances are alleviated (reduced by 26.1–72.6%) by yoga	
***Nurse-led disease management programs***					
Chen, 2016	3* (343)	KRT	KDQoL, KDQOL-SF, KDoL-36, SF-36	Sleep symptoms improved by nurse-led disease management program (MD:9.79, 95% CI 5.44, 14.15,* I*^2^: 39%)	
***Combination of non-pharmacological***					
Kesik 2023	10* (458)	HD	IRLSSG	RLS improved by mixed non-pharmacological interventions (SMD: – 1.53, 95% CI – 1.72, – 1.34, *I*^2^: 72.9%)	
Sharma, 2020	6	HD	PSQI	No definitive conclusion on the effect of a mixed pharmacological intervention including exercise, music, and muscle training, on sleep disturbances (4 primary studies showed a significant improvement, and 2 studies did not show significant improvement)	
***Combination of pharmacological***					
Natale, 2019	6 (234)	Mixed	PSQI	No definitive conclusion on pharmacological interventions to improve sleep quality due to very low-certificate evidence. Meta-analysis was not performed	
Chen, 2021	24* (1,252)	KRT	PSQI, IRLS, PLMI, RLS-6, SF-36, RLS-QoL28, RLSQ	RLS improved most by cool dialysate (MD: 16.82, 95% CI 10.63, 23.02) and gabapentin (MD: 8.95, 95% CI 1.85, 15.85)	
Gopaluni, 2016	6	Mixed	IRLSSG	No definitive conclusion on impact of pharmacological interventions on RLS due to small size and short follow-up. Meta-analysis was not performed	
**HEALTH OUTCOMES**					
***Puritis and QoL***					
Poku, 2022	20	HD	SF-36, KDQoL-SF, SF-12,	Sleep disturbance mediates the relationship between pruritus and QoL	
***Mortality***					
Yang 2018	18* (6,890)	Mixed	Choice health experience questionnaire, JESS, Activity monitor, PSQI, ESS, Sleep Heart Health Study Sleep Habits Questionnaire, AHI, oximetry, ODI, ESS, Flemmons criteria questionnaire, self-reported snoring, RDI, PSG, PLMS	Sleep disturbances increased cardiovascular events and all-cause mortality (RR:1.47, 95% CI = 1.30–1.66,* I*^2^: 59.7%)	
Puthenpura 2020	7* (186,686)	Mixed	Oximetry, ICD-9 code	Sleep apnoea increased cardiovascular events (OR: 1.02, 95% CI 0.91, 1.12,* I*^2^: 80.6%) and overall mortality (OR: 2.09, 95% CI 1.59, 2.74,* I*^2^: 0%)	
***Post Dialysis Fatigue***					
You 2022	13* (190)	HD	NR	Sleep disturbances are significantly associated with PDF. (OR: 0.24, 95% CI 0.19–0.30)	
***Cognitive Function***					
Oh 2018	39* (370)	HD	NR	Sleep disturbances significantly corrected cognitive impairment	
**DETERMINANTS OF SLEEP**					
Huang 2023	20	Pre-dialysis CKD	PSQI, KDOQoL, ISI, SHPS, PSAS, Actigraphy, ESS, Center for Epidemiologic Studies Depression Scale short form, MOSSleepR	Demographics, physiological conditions, depression, smoking, arousal-related and cognitive arousal behaviours were associated with poor sleep quality	
**PATIENT EXPERIENCE**					
Cheng 2021	48	KRT	Self-reported experience	The treatment and symptom burden of dialysis disrupts and deprives patients of sleep, which leads to overwhelming and uncontrollable exhaustion	

*AHI* Apnoea- Hypopnea Index, *AIS* Athens Insomnia Scale, *Brief COPE* Coping Orientation to Problems Experienced inventory, *CI* Confidence Intervals, *CKD* chronic kidney disease, *CKD-SBI* CKD Symptom Burden Index, *CKD-SI* Chronic Kidney Disease Symptom Index, *CSE* Chinese Symptom Experience, *DSI* Dialysis Symptom Index, *DASS-21* Depression anxiety and Stress Scale-21, *ESAS* Edmonton Symptom Assessment System, *ESS* Epworth sleepiness scale, *EQ-5D-5L* EuroQoL 5-dimentsion 5-level, *HD* haemodialysis, *ICSD* International Classification of Sleep Disorders, *ICD-9* International Classification of Diseases, *ISI* insomnia severity index, *IRLLSSG* International Restless Legs Syndrome Study Group Rating Scale, *JESS* Japanese version of the Epworth Sleepiness Scale, *KDQOL-36* kidney disease quality of life-36, *KDQoL-SF* kidney disease quality of life-Short Form, *KRT* Kidney Replacement Therapy, *LUSS* Leicester Uraemic Symptom Score, *MD* Mean Difference, *MSLT* Multiple Sleep Latency Test, *MSAS-SF* Memorial Symptom Assessment Scale—Short Form, *MOS-SPI* Medical Outcomes Study Sleep Problems Index, *MOSSleepR* medical outcomes study sleep scale–revised, *ODI* Oxygen Desaturation Index, *OR* Odds Ratio, *PD* Peritoneal Dialysis, *POS-Renal* Palliative care Outcome Scale, *PSAS* pre-sleep arousal scale, *PSQI* Pittsburgh sleep quality index, *PSG* polysomnography, *PLMS* Periodic Limb Movement Syndrome, *RDI* respiratory disturbance index, *RLS-6* RLS 6-item questionnaire, *RLS-QoL28* RLS quality-of-life questionnaire, *RR* Risk Ratio, *SDQ* Sleep Disorders Questionnaire, *SF-36* Short Form Survey, *SHPS* sleep hygiene practice scale, *SMD* Standard Mean Difference, *VAS* Visual Analog Scale, *WHO-QOL- BREF* World Health Quality of Life Abbreviated

*Meta-analysis. N: sample size in meta-analysis

^+^Studies reported several interventions

^a,b^Used in meta-analysis to distinguish data between poor sleep quality and insomnia

Three main aspects of sleep were observed: sleep apnoea (including sleep-disordered breathing), restless legs syndrome (including periodic limb movement syndrome) and other sleep disturbances (including sleep quality, poor sleep, insomnia, and total sleep time). Most reviews (72%, 36 reviews) were on other sleep disturbances, followed by seven reviews each on sleep disorders in restless legs syndrome and sleep apnoea. Five main focuses were identified in the included reviews: (1). Interventions (58%, 29 reviews), (2). Prevalence (28%, 14 reviews), (3). Health outcomes (10%, 5 reviews), (4). Determinants of sleep (2%, 1 review), and (5). Patient experience (2%, 1 review).

The study populations mainly consisted of patients on haemodialysis (HD) (44%, 22 reviews), followed by mixed stages of CKD patients (32%, 16 reviews) and a mix of kidney replacement therapy (KRT) (16%, 8 reviews). Fewer reviews examined specific groups, such as CKD not on dialysis (1 review), CKD pre-dialysis (1 review), elderly individuals (> 60 years old) with CKD receiving conservative management (1 review) and peritoneal dialysis (PD) patients (1 review). Instruments used to report sleep disturbances were not reported in three reviews [24, 34, 35]. A detailed description of the instruments is presented in Table 2.

### Prevalence

#### Sleep disturbances

**Fig. 2:**
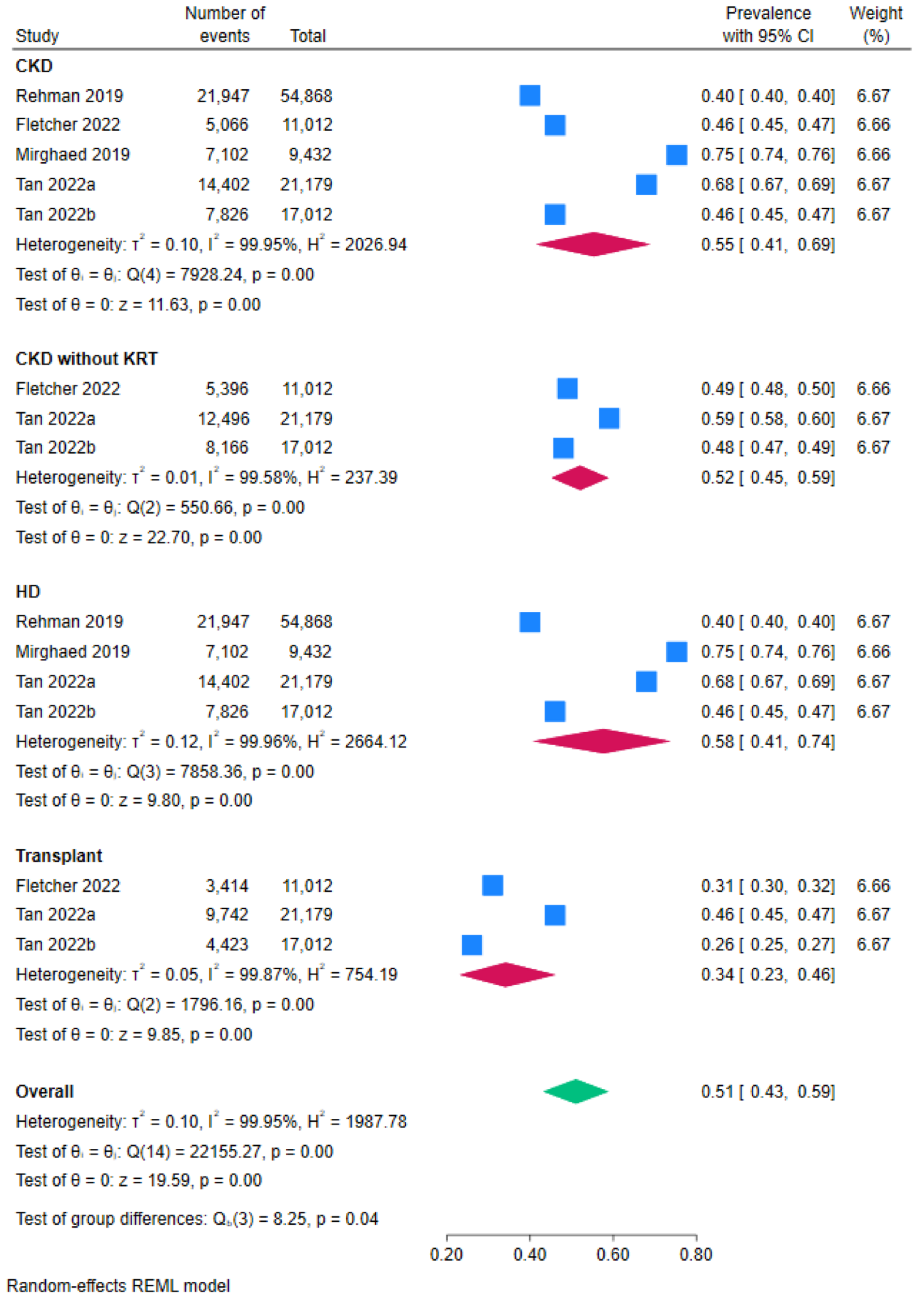
Summary pooled prevalence of sleep disturbances to different stages of CKD

The prevalence of sleep disturbances was investigated in three systematic reviews and four meta-analyses. The narrative findings from the three systematic reviews reported that sleep disturbances were one of the most prevalent symptoms in CKD patients [1], with a prevalence of over 40% [15, 36]. There was a slight study overlap among four meta-analyses (CCA: 1.55%, 129 primary studies). The pooled results from four meta-analyses [14, 37–39] demonstrated that the overall prevalence of sleep disturbance in people with CKD was 55% (95% CI 41, 69). The pooled prevalence of sleep disturbances for HD patients [14, 37, 39], CKD patients not receiving KRT [14, 38] and transplant recipients [14, 38] was 58% (95% CI 41, 74), 52% (95% CI 45, 59), and 34% (95% CI 23, 46), respectively (Fig. 2). A complete list of overlap graphs is included in Supplementary Table 3.

#### Sleep apnoea

Sleep apnoea prevalence was investigated in three meta-analyses and one systematic review. The systematic review [40] reported the prevalence of central sleep apnoea as 9.6% in people with CKD. There was a high overlap among the meta-analyses [16, 41, 42](CCA: 10.08%, 122 primary studies). Therefore, the prevalence is reported based on the most recent meta-analysis [16] that included 107 primary studies, demonstrating that the overall prevalence for CKD patients not on dialysis was 59% (95% CI 42, 71) and 49% (95% CI 47, 52) for the end-stage kidney disease population.

#### Restless legs syndrome

Three meta-analyses reported the prevalence of restless legs syndrome, and the overlap among these meta-analyses was very high (CCA: 23.08%, 127 primary studies). Therefore, the prevalence is reported based on the most recent review [17] that included 97 primary studies, demonstrating that the overall prevalence of restless legs syndrome in HD patients was 27.2%. Of the other two meta-analyses, one reported the prevalence across different stages of CKD [43], which showed a lower prevalence in the early stages of CKD (9.9%) and transplant recipients (6.7%). The prevalence of restless legs syndrome in Iranian HD patients was reported at 50% [34].

### Interventions

A total of twenty-nine reviews reported the effectiveness of interventions in improving sleep disturbances [18–20, 44–62], sleep apnoea [63, 64] and restless legs syndrome [21, 65–67]. These interventions were categorised into 12 groups of intervention namely; acupressure [18, 20, 48–50, 68, 69], exercise [18, 19, 55, 56, 65, 67, 68], aromatherapy [44, 46, 70], mindfulness [59, 60, 68], dialysis [53, 54, 63, 64], muscle relaxation [18, 58], music [57], acupuncture [51, 52], yoga [50, 61], combination of non-pharmacological [66], nurse-led disease management [62], and combination of pharmacological interventions [18, 21, 67]. Table 3 presents a summary of the results of interventions from the review and the overlap among reviews.

**Table 3:** Summary of outcomes of interventions and the overlap among reviews

Intervention	Number of reviews	Number of MA (studies in MA) *	Positive effect	No effect	Inconclusive	Overlap	Outcome (effect size)^α^	
**Sleep disturbances**								
Acupressure	7	5 (19) *	✓✓✓✓✓		✓✓	Very high	No difference (MD: – 0.51, 95% CI – 2.75, 1.73)^α^	
Exercise	4	4 (25) *	✓✓		✓✓	Very high	No difference (MD: – 1.10, 95% CI – 2.26, 0.05)	
Aromatherapy	3	1 (6)	✓✓✓			NA	Positive (SMD: – 1.52, 95% CI – 2.38, – 0.67)	
Mindfulness	3	1 (3)		✓✓	✓	NA	No difference (SMD: 0.44, 95% CI – 0.28,1.17)	
Dialysis	2	1 (16)	✓✓			NA	Positive (RR: 0.58, 95% CI 0.44, 0.64)	
Muscle relaxation	2	2 (10) *	✓✓			No overlap	Positive (MD: – 1.69, 95% CI – 1.95, – 1.43)^α^	
Yoga	2	0	✓✓			NA	Positive (no meta-analysis)	
Acupuncture	2	1 (4)	✓		✓	NA	Inconsistent (MD: – 2.46, 95% CI – 4.32, – 0.69)	
Music	1	1 (3)	✓			NA	Positive (Hedge's g: 1.95, 95% CI 0.92, 2.97)	
Nurse-led management	1	1 (3)	✓			NA	Positive (MD: 9.79, 95% CI 5.44, 14.15)	
Combination of non-pharmacological	1	0			✓	NA	Inconclusive (no meta-analysis)	
Combination of pharmacological	1	0			✓	NA	Inconclusive (no meta-analysis)	
**Sleep apnoea**								
Nocturnal dialysis	2	2 (7) *	✓✓			Very high	Positive (MD: – 11.9, 95% CI – 13.47, – 10.37)	
**Restless legs syndrome**								
Exercise	2	2 (5) *	✓✓			Very high	Positive (SMD: – 1.79, 95% CI – 2.21, – 1.37)	
Non-pharmacological interventions	1	1 (10)	✓			NA	Positive (SMD: – 1.53, 95% CI – 1.72, – 1.34)	
Combination of pharmacological intervention	2	1 (24)	✓		✓	NA	Inconsistent	

Each tick indicates a review

*MA* meta-analysis, *MD* mean difference, *NA* not applicable, *RR* risk ratio, *SMD* standard mean difference

*Indicates the number of studies included in the reviews, not counting the overlapped studies. α pooled effect size

### Sleep disturbances

#### Acupressure

The effects of acupressure on sleep quality were reported in two systematic reviews and five meta-analyses. The two systematic reviews presented inconsistent results, with one including three studies [69] and the other six studies [50](Table 2). The overlap among the five meta-analysis reviews [18, 20, 48, 49, 68]was very high (CCA: 20.90%). Consequently, the pooled effect of acupressure was calculated from two non-overlapped meta-analysis [49, 68] and a Cochrane review [18], which found no significant difference in sleep quality with acupressure (mean difference (MD): – 0.51, 95% CI – 2.75, 1.73).

#### Exercise

The effects of exercise on sleep quality were reported in four meta-analyses [18, 19, 55, 68], with a very high degree of overlap among them (CCA: 19.35%). Due to this overlap, the findings were summarised based on the Cochrane review [18], which indicated an uncertain effect of exercise on sleep quality (MD: – 1.10, 95% CI – 2.26, 0.05).

#### Aromatherapy

The effects of aromatherapy on sleep disturbances were investigated in two reviews and one meta-analysis. Both reviews [44, 46] reported improvements in sleep quality associated with the use of aromatherapy. Similarly, the meta-analysis (*n* = 6 studies) demonstrated a significant reduction in sleep questionnaire scores (Standard Mean Difference (SMD): – 1.52, 95% CI – 2.38, – 0.67), indicating a positive effect of aromatherapy on sleep quality [70].

#### Mindfulness-based interventions

The effects of mindfulness were investigated in two systematic reviews and one meta-analysis. The two systematic reviews presented inconsistent results, each including a small number of studies (n = 2 studies [59] and *n* = 1 study [60]). The meta-analysis (n = 3 studies) examined the effects of mindfulness-based interventions, such as cognitive behavioural therapy, and found no significant impact on sleep quality (SMD: 0.44, 95% CI – 0.28,1.17) [68].

#### Dialysis

The effects of dialysis were reported in a systematic review and a meta-analysis. The systematic review [53] (*n* = 4 studies) found that, compared to PD, in-centre dialysis was associated with better sleep quality. The meta-analysis [54] (*n* = 16 studies) demonstrated that higher-intensity KRT, including intensive HD, PD, or transplant, was associated with improved sleep quality (Risk ratio (RR): 0.58, 95% CI 0.44, 0.64).

#### Muscle relaxation

The effects of muscle relaxation were reported in two meta-analyses [18, 58], with no overlap between the included studies. The results were conflicting: one reported an improvement in sleep quality with progressive muscle relaxation [58]; while the other one highlighted the uncertainty in the evidence due to a wide range of confidence intervals (MD: 1.62, 95% CI – 5.03, 1.79) [18]. However, the pooled analysis of 10 studies showed a significant positive effect of muscle relaxation on sleep quality (MD: – 1.69, 95% CI – 1.95, – 1.43).

#### Yoga

The effects of yoga were reported in two systematic reviews [50, 61], both of which demonstrated positive outcomes in improving sleep quality. One review [50] included three primary studies showing that yoga improved sleep quality symptoms by 26.1–72.6%

#### Other interventions

A review of music [57] and nurse-led programs [62], also indicated improvements in sleep quality; however, the evidence was limited, with fewer than three primary studies included in each review. The effectiveness of combining non-pharmacological [56], and pharmacological [18] interventions, remains inconclusive.

### Sleep apnoea

#### Nocturnal dialysis

Two meta-analyses investigated the impact of nocturnal haemodialysis on sleep apnoea [63, 64]. Both reviews reported a significant improvement in sleep apnoea under nocturnal haemodialysis, as measured by the apnoea-hypopnea index (AHI). Due to the very high overlap between reviews (CCA: 28.6%), the effect of nocturnal dialysis was summarised based on the most recent review [64], which showed a significant reduction in apnoea-hypopnea index (MD: – 11.9, 95% CI – 13.47, – 10.37), indicating improved sleep apnoea.

### Restless legs syndrome

A total of three meta-analyses, one systematic review and one network meta-analysis investigated the interventions to improve restless legs syndrome. Two meta-analyses [65, 67] examined the effect of exercise, with a very high overlap between these studies (CCA: 20%). Therefore, the effects of exercise on restless legs syndrome were reported based on the most recent meta-analysis [65], which demonstrated that exercise significantly improved restless legs syndrome (SMD: – 1.79, 95% CI – 2.21, – 1.37). The effects of pharmacological interventions on restless legs syndrome were inconclusive [67]. However, a meta-analysis found that a combination of non-pharmacological interventions was effective for restless legs syndrome (SMD:-1.53, 95% CI – 1.72, – 1.34) [66]. A network meta-analysis indicated that among interventions, cool dialysate (MD: 16.82, 95% CI 10.63, 23.02) and gabapentin (MD: 8.95, 95% CI 1.85, 15.85) were the two most effective interventions for restless legs syndrome [21].

### Health outcomes

Five reviews (97 primary studies) reported health outcomes associated with sleep disturbances [23, 24, 35, 71] and sleep apnoea [22]. These reviews suggest that sleep disturbances are associated with cognitive impairment [35], post-dialysis fatigue [24] and increased mortality (RR: 1.47, 95% CI 1.30–1.66) [23], and is a link between pruritus and QoL [71]. Increased cardiovascular events (Odds Ratio: 1.02, 95% CI 0.91, 1.12) and overall mortality (Odds Ratio: 2.09, 95% CI 1.59, 2.74) [22] were also observed in patients with CKD and co-existing sleep apnoea. The overlap analysis was not performed in health outcomes as each review assessed different outcomes.

## Discussion

This review aims to assess the existing evidence on sleep disturbances and sleep disorders in the adult CKD population and identify prioritised areas for future research. The present review demonstrated a high prevalence of sleep disturbances and sleep disorders, including sleep apnoea and restless legs syndrome, among people with CKD. There has been extensive and rapidly growing research on interventions. While some interventions (e.g., aromatherapy, dialysis, muscle relaxation, yoga and music) have demonstrated a positive impact on sleep quality, others (e.g., acupressure, exercise, mindfulness, acupuncture and pharmacological interventions) show uncertain or no significant effect. Despite a large number of studies on the effectiveness of these interventions, the high overlap among reviews made drawing significant conclusions on the effects of interventions challenging. Additionally, we found that despite a robust search and a wide range of reviews on this topic, there was a paucity of studies exploring the determinants of sleep and patient experience of sleep disturbances, highlighting a gap for future research.

This umbrella review showed overwhelming evidence regarding the high prevalence of sleep disturbances, sleep apnoea, and restless legs syndrome in the CKD population. Despite the high overlap among some reviews in sleep apnoea and restless legs syndrome, more than 100 primary studies were involved in generating evidence for each aspect of sleep disturbances. This information shows the burden of sleep problems in the CKD population and supports the notion of the recent Kidney Disease Improving Global Outcomes (KDIGO) controversies conference (2023) consensus that screening sleep symptoms should be initiated within the kidney care team as a first step to support the management of symptom burdens in CKD patients [72]. Several mechanisms have been proposed to explain the association between sleep disturbances in people with kidney disease, including the accumulation of uraemic toxins and systemic inflammation, along with consequent changes in biochemical parameters, such as altered melatonin secretion, all leading to sleep–wake disturbances [73]. These pathophysiological links may explain the high prevalence of sleep disturbances in the CKD cohort. Despite a slightly lower prevalence in the transplant cohort, the pooled prevalence still indicated that 34% of transplant recipients experience sleep disturbances; interventions to improve sleep disturbances in this cohort are warranted.

We found a significant number of reviews (29 reviews) that focused on interventions to improve sleep disturbances. While interventions such as aromatherapy, muscle relaxation, music, yoga, dialysis and nurse-led programs demonstrated promising effects on improving sleep quality, the evidence was limited by the small number of supporting studies, restricting the generalisability of the results. Dialysis and nocturnal haemodialysis showed significant benefits for sleep quality and sleep apnoea, respectively, highlighting the importance of tailored therapeutic approaches in specific patient populations. However, the feasibility of nocturnal haemodialysis and switching to a different dialysis modality requires further examination. For restless legs syndrome, exercise and certain non-pharmacological strategies, including cool dialysate and gabapentin, showed effectiveness, though this was based on a single meta-analysis and network meta-analysis. The lack of conclusive evidence can be attributed to the high overlap in the meta-analysis. The high degree of overlap among studies, particularly in several meta-analyses, significantly limits the strength and reliability of the findings, resulting in inconclusive evidence. This overlap reduces the ability to draw definitive conclusions about the efficacy of certain interventions, as it increases the risk of duplication and bias in the pooled analyses. Consequently, the true effects of these interventions remain uncertain, underscoring the need for further studies to provide conclusive evidence. Nevertheless, the results of this umbrella review highlight the potential benefits of integrating some non-pharmacological interventions into mainstream medicine. These findings underscore the need for robust, high-quality research to better define the efficacy and applicability of these interventions in enhancing sleep health. Future studies examining how these interventions can be adopted and embedded into the lifestyle of CKD patients to improve their quality of life are also needed.

To mitigate sleep disturbances, it is crucial to identify the determinants of sleep. Determinants of sleep in adults can be broad and complex, including biological factors such as age and sex, as well as behavioural factors like alcohol consumption or caffeine intake [74]. A recent publication from the Global Sleep Health Task Force emphasises the importance of recognising environmental and social determinants of sleep, such as sleep environments, lighting and noise [75]. In this review, we found only one systematic review focused on sleep determinants and one that explored the issue of sleep from the patient’s perspective. Considering the complexity of sleep, future research should aim to understand patients’ perspectives on sleep. Initial efforts may involve qualitative studies to understand sleep determinants from the patients’ perspectives. This understanding will assist in assessing the suitability of current sleep education guidelines and designing future interventions to embed non-pharmacological interventions in the CKD population.

No previous attempt has been made to conduct an overview summarising all available evidence on the complex sleep issues in the CKD population. This approach assessed the quality of the current research landscape and identified gaps to avoid unnecessary waste of time and resources conducting reviews on well-known areas. Other key strengths include the systematic search methodology, the use of appraisal tools, and overlap analysis to provide a high-level summary of evidence. However, the decision to include systematic reviews exclusively may have resulted in the omission of recently published papers, which should be considered a limitation in this review. Additionally, although some systematic reviews may be methodologically sound, they were based on small primary studies, which must be considered when interpreting results. Furthermore, the quality of primary studies included in the systematic review was not assessed. Lastly, there is no consensus regarding the best way to manage overlap in meta-analyses; therefore, this review could not present the overall pool effect size for each intervention. However, the intent of our review is to provide a synthesised summary of existing research for policy or clinical decision-makers to understand the quality of evidence and prioritised areas for future research.

## Conclusions

This umbrella review highlights the high prevalence of sleep disturbances in people with CKD, as supported by extensive systematic reviews and meta-analyses. The literature suggests that non-pharmacological interventions such as aromatherapy, higher-intensity dialysis, and muscle relaxation can improve sleep quality. Nocturnal dialysis and exercise can improve sleep apnoea and restless legs syndrome, respectively. However, the small number of primary studies included in meta-analyses limits the strength of the evidence. Additionally, the high overlap among the reviews increases the risk of bias, which is a drawback of the current literature, leaving many interventions inconclusive. Robust research is needed to establish the most effective interventions for sleep disturbances. This review also identifies a gap in understanding the determinants of sleep from patients’ perspectives, which is crucial for developing appropriate strategies to improve sleep in individuals with CKD. Future studies should focus on addressing these gaps.

## Data Availability

All data generated or analysed during this study are included in this published article and its supplementary information files.
